# ASO Author Reflections: Locally Recurrent Rectal Cancer from a Nationwide Perspective

**DOI:** 10.1245/s10434-023-13207-x

**Published:** 2023-02-10

**Authors:** Hidde Swartjes, Tijmen Koëter

**Affiliations:** 1 Department of Surgery, Radboud Institute for Health Sciences, Radboud university medical center, Nijmegen, The Netherlands; 2 Department of Surgery, Elisabeth-TweeSteden Ziekenhuis, Tilburg, The Netherlands

## Past

Locally recurrent rectal cancer (LRRC) is a commonly studied outcome after rectal cancer surgery, in contrast with the less frequently studied locoregional recurrence of colon cancer.^1^ The broad implementation of the total mesorectal excision (TME) in the 90s initiated a great reduction of local recurrence rates. Combined with high-quality magnetic resonance imaging (MRI) and neoadjuvant treatment strategies, such as (chemo)radiotherapy, rectal cancer treatment has become increasingly effective. So effective even, that the recommendation for neoadjuvant radiotherapy for early-stage rectal cancer was omitted from several nationwide guidelines in Western Europe, due to its low added benefit. In the seemingly ever-changing field of rectal cancer treatment, population-based studies can be of great value. They provide a robust state-of-the-art overview of current disease treatments between the various frequently occurring single- and multi-center retrospective cohort studies. Unfortunately, population-based studies on LRRC incidence, treatment, and outcomes are scarce.

## Present

The results from our nationwide, population-based, retrospective cohort study reflect a 3-year cumulative incidence of LRRC of 6.4% among 1431 patients who were diagnosed with primary rectal cancer in the second semester of 2015 and subsequently treated with TME surgery.^2^ This incidence rate was comparable with previously conducted studies from the TME era. Distant metastases coincided with LRRC in 42.9% of patients diagnosed with a local recurrence. Previously known risk factors for LRRC diagnosis [i.e., distal localization, R1-2, (y)pT3-4 and (y)pN1-2] were confirmed by our multivariable analysis. Curative-intent treatment was given to 43% of patients with LRRC, which was a higher proportion than in previous population-based studies.^3,4^ Moreover, the 3-year overall survival estimate for patients treated with curative intent (70%) was surprisingly high. Additionally, our study affirmed that patients who did, versus patients who did not, receive prior neoadjuvant (chemo)radiation for their primary rectal cancer, were equally often treated with neoadjuvant (chemo)radiation for their local recurrence.

## Future

The state-of-the-art overview provided by our study reflects very respectable outcomes for the treatment and overall survival of LRRC in the Netherlands. Several factors have presumably contributed to these outcomes. The increased centralization of care for patients with LRRC induced more specialized knowledge. This knowledge is likely of great value in the multidisciplinary team meetings, in which the treatment intention of patients with LRRC is decided. Finally, ongoing initiatives are enforcing further improvement of the treatment of patients with LRRC. For example, the current PelvEx II study will assess the effect of induction chemotherapy in patients with LRRC undergoing neoadjuvant chemoradiation, prior to the resection of the local recurrence.^5^ Results are to be awaited, but might contribute to treatment of LRRC becoming “a second chance at cure.”
